# Channel Phase Calibration for High-Resolution and Wide-Swath SAR Imaging with Doppler Spectrum Sharpness Optimization

**DOI:** 10.3390/s22051781

**Published:** 2022-02-24

**Authors:** Man Zhang, Sha Huan, Zeya Zhao, Zhibin Wang

**Affiliations:** 1 School of Electronic and Communication Engineering, Guangzhou University, Guangzhou 510006, China; manzhang401@gzhu.edu.cn; 2 Beijing Institute of Tracking Telementry & Telecommunication, Beijing 100094, China; zzy_kelly@163.com; 3 Beijing Institute of Remote Sensing Satellite, Beijing 100854, China; wzblq198905@163.com

**Keywords:** synthetic aperture radar (SAR), high resolution and wide swath (HRSW), phase calibration, maximum sharpness optimization

## Abstract

Channel phase calibration is a crucial issue in high resolution and wide swath (HRWS) imagery with azimuth multi-channel synthetic aperture radar (SAR) systems. Precise phase calibration is definitely required in reconstructing the full Doppler spectrum for precise HRWS imagery without high-level ambiguities. In this paper, we propose a novel calibration for HRWS SAR imagery by optimizing the reconstructed unambiguous Doppler spectrum. The sharpness of the reconstructed Doppler spectrum is applied as the metric to measure the unambiguity quality, which is maximized to retrieve the element phase error caused by channel imbalance. Real data experiments demonstrate the performance of the proposed calibration for ambiguity suppression in HRWS SAR imagery.

## 1. Introduction

Synthetic aperture radar (SAR) techniques require generating imagery with simultaneous high resolution and wide swath (HRWS) capability. However, in order to receive unambiguous echoes in range or/and Doppler, conventional SAR observed swath should be restricted by the fundamental trade-off between Doppler ambiguity and spatial resolution for a single-channel radar system [1]. With the assistance of an azimuth multi-channel system configuration, this basic restriction can be overcome by digital beamforming (DBF) techniques [2,3,4] on multiple receiver channel data. The received signal of each channel is simultaneously recorded and digitalized. By a Doppler spectrum reconstruction with DBF processing, the unambiguous and full Doppler spectrum can be retrieved to generate the HRWS SAR imagery, which systematically breaks the fundamental trade-off of azimuth resolution and range swath in a single channel SAR system. In general, these multi-channel HRWS SAR systems [5,6,7] can be sorted into multiple antenna systems and distributed systems. Several novel DBF algorithms have also been proposed for resolving Doppler ambiguity in the spatial domain [8,9,10,11,12,13,14,15] for these systems. Azimuth multi-channel SAR with DBF is naturally an effective approach to obtain unambiguous HRWS imagery in practice. However, unavoidable channel errors would significantly degrade the performance of ambiguity suppression, especially for an observing scene with strong targets, such as a strong ship under ocean circumstances. Channel mismatch calibration is crucial for HRWS imagery in azimuth multi-channel SAR systems.

Due to unexpected systematic and circumstance factors, such as mismatched transmitting and receiving channel characteristics. Channel imbalance brings additional phase errors between multiple channels; these errors degrade the DBF Doppler spectrum reconstruction performance dramatically. Before applying the receiving beamforming technique for Doppler spectrum reconstruction, the multi-channel SAR system should be well calibrated by compensating for the phase and amplitude imbalance between channels. In particular, the phase errors cause unexpected spectrum folding and mismatch in the DBF process, leaving serious ambiguities in the SAR imagery. Their presence will deteriorate the azimuth ambiguity-to-signal ratio (AASR) [16] of the HRWS imagery. In general, the imbalance phase errors are expected to be well compensated by calibration techniques. Recently, a series of innovative calibration algorithms [17,18,19,20,21,22,23] have been developed, which are roughly classified into two sorts, including inner calibration and data-driven calibration. In the inner calibration methods, the azimuth channel imbalance is expected to be compensated by an extra antenna measurement system [18,23], which would limit its real applications due to increased system complexity. On the other hand, data-driven calibration algorithms try to estimate the channel imbalance from data itself in an adaptive manner without any increase in system complexity. In this paper, we focus only on data-driven calibration for HRWS SAR imagery. Among the data-driven calibration algorithms, subspace projection algorithms [16,17,20,21] have been well investigated in many works. They generally estimate the channel phase errors for multi-channel SAR HRWS imagery with the same basic principle. The covariance matrix in the range-Doppler domain was expected to be estimated from the data itself. Then, eigenvalue decomposition on the covariance matrix was followed to separate the signal subspace and noise subspace. The signal subspace was spanned by the signal steering vectors with phase errors, which are orthogonal to the noise subspace. By minimizing the projection of the estimated steering vectors on the noise subspace, the imbalance phase errors are obtained. Typically, in reference [16], an auto-calibration algorithm developed on the virtual-source calibration was presented to estimate the channel errors for the distributed multi-receive radar configuration. The multi-channel data in the azimuth time domain is transferred into the Doppler domain via Fourier transform, and signal elements corresponding to different directions within each Doppler bin are regarded as the correction virtual sources. In subspace projection kind algorithms, by an iterative optimization projection into the noise subspaces, the element phase errors are expected to be obtained. Subspace projection-based channel calibrations are very popular in HRWS imaging with multi-channel SAR systems. In these works, the imbalance phase errors are usually assumed to be spatial-invariant. However, the extension to the azimuth- and range-dependent imbalance calibration is not hard by introducing the local invariant assumption and using local covariance matrix decomposition processing in the range-Doppler domain. It is necessary to emphasize that, in the subspace channel calibrations, an additional spatial degree of freedom is required, which means the channel number should be larger than that of the ambiguity. This requirement constrains both the azimuth multi-channel system design and HRWS maximum performance, which motivates our work in this paper.

In this paper, we developed a sharpness optimization-based phase calibration for HRWS SAR imagery with azimuth multi-channel configuration. In the algorithm, the imbalance phase error is assumed to be spatial-invariant. Based on the fact that the reconstruction of the full Doppler spectrum depends on the channel phase errors, the signal energy of the reconstructed Doppler spectrum will not separate into different ambiguous bins presenting sharp formation in the full Doppler domain. In the azimuth phase calibration, the estimation of the channel phase errors turns into maximizing the sharpness of the reconstruction full Doppler spectrum by correcting phase errors within ambiguity resolving, which simplifies the problem of calibration due to no need for additional spatial freedom degrees. In contrast to the former approaches, this method proposed in this paper calibrates phase imbalance in a robust manner by using all range bins as calibration sources. In addition, we can obtain the global optimal solution efficiently by accelerating the optimization with some useful skills. Detailed analysis of its performance is presented by utilizing a real SAR dataset.

This paper is organized as follows. In Section 2, the Doppler ambiguity signal model and the inverse filtering for ambiguity removal are constructed. Section 3 extends to the proposed channel phase calibration. Section 4 evaluates the comparison performance of the approach. Finally, some conclusions are drawn.

## 2. HRWS Imaging with Azimuth Multi-Channel SAR Configuration

In this section, we introduce the azimuth multi-channel SAR for HRWS imagery generation and the Doppler ambiguity resolving with inverse-filtering processing.

### 2.1. Azimuth Multi-Channel SAR Mathematical Model

**Figure 1:**
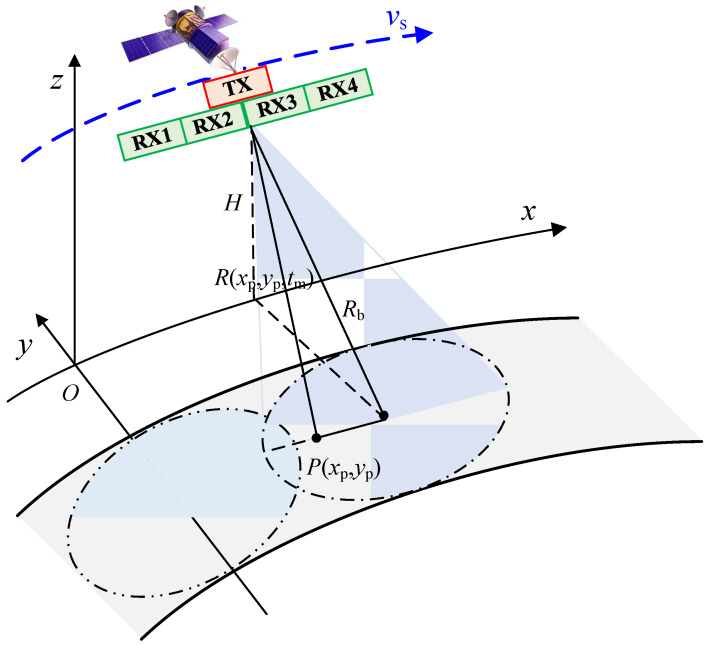
Geometry of azimuth multi-channel SAR.

The azimuth multi-channel SAR geometry is shown in Figure 1. The reference equivalent phase center (EPC) is at the origin of the Cartesian coordinate. The platform moves along the x-axis at velocity $v_{s}$. Usually, the system uses a reference antenna to transmit a wide-beam signal, and all antennas receive returns simultaneously. The data can be converted into the equivalent self-transmitting and self-receiving data by compensating a constant phase with respect to the reference channel [9]. In the ideal model, the channels are supposed to be calibrated and the baseline between equivalent phase centers is constant during the coherent processing interval (CPI). Assume that *m* = 1, 2, …, *M* and *M* is the number of equivalent phase centers. The mth equivalent phase center is $\left(x_{m},0,0\right)$ at the time t=0 and $\left(x_{m}+v_{s}t,0,H\right)$ at time t. Referring to [17], the echo received at the mth EPC at time t can be written as follows:

(1) $$\begin{array}{cc} s_{c,m}\left(\tau ,t\right)= & \iint \sigma \left(x,y\right)\cdot h\left[\tau -\frac{2r_{c,m}\left(x,y,t\right)}{c}\right]\cdot g\left(t-\frac{x-x_{m}}{v_{s}}\right)\\ & \cdot \exp\left[-j4\pi \frac{r_{c,m}\left(x,y,t\right)}{\lambda }\right]dxdy \end{array}$$

where

(2) $$R\left(x,y,t\right)=\sqrt{{\left(x-x_{m}-v_{s}t\right)}^{2}+R_{b}^{2}}$$

(3) $$h\left(\tau \right)=a\left(\tau \right)\cdot \exp\left(-j\pi \alpha \tau^{2}\right)$$

and α is the chirp rate; t and τ denote the azimuth slow-time and range fast-time respectively; $R_{b}^{2}=\sqrt{y^{2}+H^{2}}$; c is the propagation velocity; σ(x,y) is the clutter complex reflectivity of scatter (x,y) on ground; g(t) represents the antenna pattern and other slow time-variant characters.

After transforming (1) into the two-dimension (2D) frequency domain via a 2D Fourier transform (FT), we can obtain the following equation by using the concept of instantaneous wavenumber:

(4) $$S_{c,m}\left(K_{r},f_{d}\right)=s\left(K_{r},f_{d}\right)\cdot \exp\left(-j2\pi \frac{x_{m}}{v_{s}}f_{d}\right)$$

where

(5) $$\begin{array}{l} s\left(K_{r},f_{d}\right)=\iint \sigma \left(x,y\right)\cdot H\left(K_{r}\right)\cdot G\left(f_{d}\right)\\ \;\cdot \exp\left(-jR_{b}\sqrt{K_{r}^{2}-{\left(2\pi f_{d}\right)}^{2}/v_{s}^{2}}\right)\cdot \exp\left(-j2\pi f_{d}\frac{x}{v_{s}}\right)dxdy \end{array}$$

and $H\left(K_{r}\right)=FT\left[h\left(\tau \right)\right]$. As a result, there is only one linear phase difference $\exp\left(-j2\pi \frac{x_{m}}{v_{s}}f_{d}\right)$ between the mth and the reference EPC for the same Doppler bin output. In the Doppler domain, for a single Doppler bin containing all clutter echoes from the same azimuth angle θ. The relation between the Doppler frequency $f_{d}$ and the corresponding azimuth angle θ is given by

(6) $$f_{d}=\frac{2v_{s}\sin\theta }{\lambda }$$

**Figure 2:**
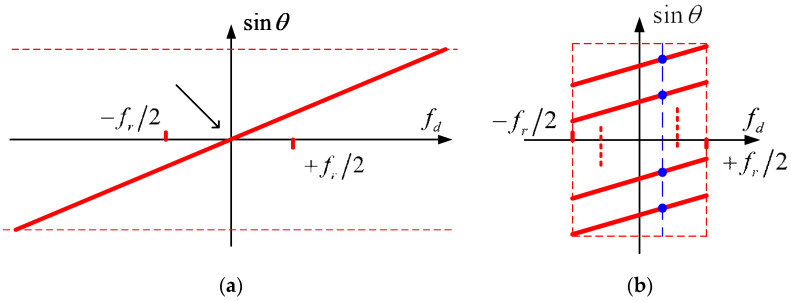
Spatial-time spectrum (**a**) without Doppler ambiguity and (**b**) with Doppler ambiguity.

This linear relationship between $f_{d}$ and sinθ can be represented in the spatial-time spectrum, as Figure 2 shows.

In the HRWS SAR imaging, the pulse repetition frequency (PRF) $f_{r}$ is usually much lower than the instantaneous Doppler bandwidth. Thus, the Doppler spectrum is ambiguous, as shown in Figure 2b, where the dots represent signal components from different directions within the identical Doppler bin. We rewrite (4) as follows:

(7) $$S_{c,m}\left(K_{r},f_{d}\right)=\sum_{i=-N+1}^{N}s\left(K_{r},f_{d}+i\cdot f_{r}\right)\cdot \exp\left[-j2\pi \frac{x_{m}}{v_{s}}\left(f_{d}+i\cdot f_{r}\right)\right]$$

where the total summation term 2N representing the Doppler ambiguity number, should not be more than the number of EPC, which is a basic constraint for all Doppler resolving algorithms, and $-f_{r}/2\leq f_{d}\leq +f_{r}/2$. For expression simplicity, we define the ambiguity number as an even number, which does not affect handling ambiguity resolving for odd cases. Therefore, the array steering matrix for the Doppler bin $f_{d}$ is given by

(8) $$A\left(f_{d}\right)=\left[\begin{array}{ccccc} a_{-N+1}, & \cdot \cdot \cdot & a_{i}, & \cdot \cdot \cdot & a_{N} \end{array}\right]$$

The array steering vector for the Doppler component $f_{d}+i\cdot f_{r}$ is

(9) $$a_{i}={\left[\begin{array}{ccccc} 1, & \cdot \cdot \cdot & \exp\left(-j2\pi \frac{x_{m}}{v_{s}}\left(f_{d}+i\cdot f_{r}\right)\right), & \cdot \cdot \cdot & \exp\left(-j2\pi \frac{x_{M}}{v_{s}}\left(f_{d}+i\cdot f_{r}\right)\right) \end{array}\right]}^{T}$$

where ${\left[\cdot \right]}^{T}$ denotes the vector transpose operator. The array output of the Doppler bin can be written as follows:

(10) $$\begin{array}{c} S_{c}\left(f_{d}\right)={\left[\begin{array}{ccccc} S_{c,1}\left(K_{r},f_{d}\right), & \cdot \cdot \cdot & S_{c,m}\left(K_{r},f_{d}\right), & \cdot \cdot \cdot & S_{c,M}\left(K_{r},f_{d}\right) \end{array}\right]}^{T}\\ S_{c}\left(f_{d}\right)=A\left(f_{d}\right)s\left(f_{d}\right)+e\left(f_{d}\right) \end{array}$$

where $s\left(f_{d}\right)={\left[\begin{array}{ccccc} s\left(K_{r},f_{d}+\left(-N+1\right)\cdot f_{r}\right), & \cdots & s\left(K_{r},f_{d}+i\cdot f_{r}\right), & \cdots & s\left(K_{r},f_{d}+N\cdot f_{r}\right) \end{array}\right]}^{T}$ is the signal vector, and $e\left(f_{d}\right)$ denotes the additive noise vector, which is assumed to be independent and white noise. The problem now is to unfold the spatial-time spectrum and reconstruct the full Doppler bandwidth spectrum.

### 2.2. Unambiguous Doppler Spectrum Reconstruction with Inverse-Filtering

In this subsection, we introduce non-adaptive beamforming for Doppler ambiguity resolving [10], which is seamless with the following channel calibration we proposed in this paper. The Doppler spectrum of (7) is divided into many short bins, and each bin corresponds to 2N Doppler components from corresponding azimuth angles. The non-adaptive beamforming approach first extracts these spectrum components from each Doppler bin by means of spatial filtering. By rearranging the extracted Doppler components in order, the full Doppler spectrum can be reconstructed. Considering no phase error involved, the spatial filtering process for Doppler ambiguity resolving is given by the following equations [16].

(11) $$\begin{array}{l} \hat{s}\left(f_{d}\right)=W^{H}\left(f_{d}\right)\cdot S_{c}\left(f_{d}\right)\\ \;\;\;\;\;\;\;\;\approx W^{H}\left(f_{d}\right)A\left(f_{d}\right)s\left(f_{d}\right) \end{array}$$

where $\hat{s}\left(f_{d}\right)$ is the reconstructed Doppler spectrum, ${\left[\cdot \right]}^{H}$ denotes the conjugation transpose operator, and the spatial filtering for the 2N Doppler components is given by

(12) $$\begin{array}{l} W^{H}\left(f_{d}\right)=H\cdot A^{-1}\left(f_{d}\right)\\ \;\;\;\;\;\;\;\;\;\;\;\;={\left[w\left(f_{d}+\left(-N+1\right)\cdot f_{r}\right),\cdots ,w\left(f_{d}+i\cdot f_{r}\right),w\left(f_{d}+N\cdot f_{r}\right)\right]}_{M\times 2N}^{H} \end{array}$$

(13) $$H=\mathrm{diag}{\left\{1,1,\cdots ,1\right\}}_{2N\times 2N}$$

It can be noted that beamforming filtering requires the inverse matrix of the steering matrix $A(f_{d})$, which means the channel number M should be not smaller than the number of ambiguity Doppler components 2N. After all the spectrum components are extracted, we rearrange them and obtain the unambiguous full spectrum. Then, conventional SAR imaging approaches can be used to focus the wide-swath image with high resolution. It is necessary to emphasize that the adaptive beamforming technique [18] usually provides better performance under non-uniform sampling than the non-adaptive beamforming Doppler ambiguity resolving in (11), in the circumstances of no imbalance phase errors involved. The adaptive beamforming filter can automatically match the interference subspaces even with the phase imbalance to generate optimal matches by using the coherence matrix decomposition. Herein, we use the non-adaptive beamforming filter to implement the phase error estimation in the following section. It is noted that the non-adaptive filter is fixed and independent of the imbalance phase errors. Non-adaptive filtering is sensitive to the phase imbalance, which can provide ideal Doppler spectrum reconstruction only when the phase imbalance is accurately corrected. This sensitivity would be helpful to develop the sharpness optimization to retrieval the channel phase errors. In real applications, after phase calibration, adaptive beamforming can be adapted to achieve optimal performance.

Now, we introduce the imbalance phase error into the signal model to pave the way for developing the following calibration optimization. As this work only focuses on the imbalance phase errors between azimuth multi-channels, by introducing the phase error matrix $\Gamma_{e}\left(\varepsilon \right)$, the multi-channel signal model in (10) can be rewritten by the following equation:

(14) $${\tilde{S}}_{c}\left(f_{d};\varepsilon \right)=\Gamma_{e}\left(\varepsilon \right)A\left(f_{d}\right)s\left(f_{d}\right)+e\left(f_{d}\right)$$

where the phase error matrix is a diagonal matrix represented as

(15) $$\begin{array}{l} \Gamma_{e}\left(\varepsilon \right)=\mathrm{diag}\left[\exp\left(j\varepsilon \right)\right]\\ \;\;\;\;\;\;\;\;\;\;\;\;=\mathrm{diag}\left[\exp\left(j\varepsilon_{1}\right),\exp\left(j\varepsilon_{2}\right),\cdots ,\exp\left(j\varepsilon_{M}\right)\right] \end{array}$$

where $\Gamma_{e}\left(\varepsilon \right)$ denotes the imbalance phase error matrix with $\varepsilon =\left[\varepsilon_{i}\right],\left(i=1,2,\cdots ,M\right)$ as the phase error vector.

Taking the imbalance phase errors into Doppler ambiguity resolving with the non-adaptive filtering in (11), the reconstructed Doppler signal depends on the phase error given by

(16) $$\begin{array}{l} \hat{s}\left(f_{d};\varepsilon \right)=W^{H}\left(f_{d}\right)\cdot {\hat{\Gamma }}_{e}^{-1}\cdot {\tilde{S}}_{c}\left(f_{d};\varepsilon \right)\\ \;\;\;\;\;\;\;\;\;\;\approx W^{H}\left(f_{d}\right)\cdot {\hat{\Gamma }}_{e}^{-1}\cdot \Gamma_{e}\cdot A\left(f_{d}\right)s\left(f_{d}\right) \end{array}$$

It is notable from (14)–(16) that, in the presence of imbalance phase errors, Doppler ambiguity removal with spatial filtering would fail. The serious mismatch between the ideal and real steering vectors will induce not only signal loss but also significant ambiguity residue. In the next section, the phase calibration to estimate $\Gamma_{e}\left(\varepsilon \right)$ will be introduced in detail.

## 3. Channel Phase Calibration with Maximum Sharpness Optimization

By optimizing the pattern design of the SAR system, the unambiguous Doppler spectrum has relative focused intensity around the Doppler centroid, while the intensity decreases away from the centroid. Due to the presence of channel phase errors, the distribution of the multi-channel reconstructed azimuth Doppler spectrum is unfocused due to the Doppler ambiguous components folding and smearing. Compared with the ideal multi-channel reconstructed Doppler spectrum, the reconstructed Doppler distribution contaminated by channel phase errors will be spread flat. Therefore, channel phase errors will lead to the broadening of the reconstructed Doppler spectrum after ambiguity removal filtering. This intrinsic phenomenon paves the way for estimating the phase errors by measuring the reconstructed Doppler spectrum distribution. At first, the reconstructed Doppler spectrum intensity is defined as follows.

(17) $$\begin{array}{l} I\left(f_{d};\varepsilon \right)={\left|\hat{s}\left(f_{d};\varepsilon \right)\right|}^{2}\\ \;\;\;\;\;\;\;\;\;\;\;\;\;\;=\hat{s}\left(f_{d};\varepsilon \right)\cdot {\hat{s}}^{*}\left(f_{d};\varepsilon \right) \end{array}$$

To sum up all reconstructed Doppler components, the sharpness function [24,25] of the reconstructed Doppler spectrum intensity is introduced as the metric of the reconstruction with the phase error as its variable.

(18) $$P\left(\varepsilon \right)=\sum_{f_{d}}I{\left(f_{d};\varepsilon \right)}^{2}$$

Herein, the sharpness optimization for phase calibration can be exploited by maximizing the sharpness of the reconstructed Doppler spectrum. The maximum sharpness optimization is given by

(19) $$\hat{\varepsilon }=\arg\;\;\;\;\max\;P\left(\varepsilon \right)$$

Herein, we have to emphasize that some other metric function can also be introduced to represent the intensity distribution property of the reconstructed Doppler spectrum, such as entropy in [26] and contrast in [27]. The reason we select sharpness mainly lies in its convenience in deriving a gradient-based solver. Newton’s solver is convenient for obtaining an optimal estimation with high efficiency [28]. This solver is implemented in an iterative manner, and we assume in the (*k* + 1)th iteration, the phase error vector is updated with the gradient vector, and the Hessian matrix is given by

(20) $$\begin{array}{l} \hat{\varepsilon }\left(k+1\right)=\hat{\varepsilon }\left(k\right)+\Delta \varepsilon_{k}\\ \;\;\;\;\;\;\;\;\;\;\;\;\;\;=\hat{\varepsilon }\left(k\right)+\left[-H^{-1}\left(\varepsilon \right)\cdot \frac{\partial P\left(\varepsilon \right)}{\partial \varepsilon }\right] \end{array}$$

where $H^{-1}\left(\varepsilon \right)$ denotes the Hessian inverse of the optimization function with respect to the phase error vector. We also introduce a variable ${\hat{\Gamma }}_{e}=\mathrm{diag}\left\{\exp\left[j\hat{\varepsilon }\left(k\right)\right]\right\}$ to denote the estimated phase error matrix in the last iteration. In addition, the phase compensation process applies ${\hat{\Gamma }}_{e}^{-1}$ on the multi-channel signal via ${\hat{\Gamma }}_{e}^{-1}\cdot {\tilde{S}}_{c}\left(f_{d};\varepsilon \right)$. The first-order gradient is expressed by the following derivations:

(21) $$\frac{\partial P\left(\varepsilon \right)}{\partial \varepsilon }=\sum_{k,r}2\cdot I\left(f_{d};\varepsilon \right)\cdot \frac{\partial I\left(f_{d};\varepsilon \right)}{\partial \varepsilon }$$

(22) ∂I(fd;ε)∂ε=∂|s^(fd;ε)|2∂ε=2⋅Re{s^∗(fd;ε)⋅∂s^(fd;ε)∂ε}

where

(23) $$\frac{\partial \hat{s}\left(f_{d};\varepsilon \right)}{\partial \varepsilon }={\left[W^{H}\left(f_{d}\right)\cdot {\hat{\Gamma }}_{e}^{-1}\cdot {\tilde{S}}_{c}\left(f_{d};\varepsilon \right)\right]}^{*}$$

and the Hessian matrix of the sharpness optimization Hessian can be derived by the following expressions:

(24) $$\begin{array}{l} H\left(\varepsilon \right)=\frac{\partial^{2}P\left(\varepsilon \right)}{\partial \varepsilon_{i}\partial \varepsilon_{j}}=\sum_{f_{d}}2\cdot I\left(f_{d};\varepsilon \right)\frac{\partial I\left(f_{d};\varepsilon \right)}{\partial \varepsilon_{i}}\\ \;\;\;\;\;\;\;\;\;\;\;\;\;\;\;\;\;\;\;\;\;\;\;\;\;\;\;=\sum_{f_{d}}2\cdot \left[\frac{\partial I}{\partial \varepsilon_{j}}\frac{\partial I}{\partial \varepsilon_{i}}+I\left(f_{d};\varepsilon \right)\cdot \frac{\partial^{2}I}{\partial \varepsilon_{i}\partial \varepsilon_{j}}\right] \end{array}$$

(25) ∂2I(fd;ε)∂εi∂εj=∂2|s^(fd;ε)|2∂εi∂εj              =2⋅Re{(∂s^∂εi)∗∂s^∂εi+s^*(fd;ε)∂2s^∂εi∂εj}

where

(26) $$\frac{\partial^{2}\hat{s}}{\partial \varepsilon_{i}\partial \varepsilon_{j}}=-W^{H}\left(f_{d}\right)\cdot {\hat{\Gamma }}_{e}^{-1}\cdot {\tilde{S}}_{c}\left(f_{d};\varepsilon \right)$$

In general, only with several iterations can the maximum of sharpness optimization be achieved. The convergence can be controlled by setting a maximum iteration number or judging the estimate difference between two sequential iterations. One can note that, in solving the optimization in (20), we need to calculate the first-order and second-order partial derivatives in each iteration. The Hessian matrix inverse determining the search step is calculated for every iteration, which involves the majority of the expensive computational load. In order to accelerate the solver to the sharpness optimization for the phase error matrix, we also give an accelerating scheme. By considering the termination condition to ensure the convergence property, it is possible to achieve a balance between the searching step accuracy and optimization efficiency. According to the principle of the BFGS (Broyden, Fletcher, Goldfard and Shanno) [29] algorithm, the Hessian matrix can be calculated approximately by an updated formula. In this manner, only the first initial direct calculation of Hessian and its inverse is calculated, and in the following iteration, Hessian can be obtained by an updating manner. This update can be given by the following equations:

(27) $$\begin{array}{ll} H_{k+1} & =H_{k}-\frac{H_{k}s_{k}s_{k}^{T}H_{k}}{s_{k}^{T}H_{k}s_{k}}+\frac{y_{k}y_{k}^{T}}{y_{k}^{T}y_{k}}\\ s_{k} & ={\hat{\varepsilon }}_{k+1}-{\hat{\varepsilon }}_{k}\\ y_{k} & =\frac{\partial P\left(\varepsilon ;k+1\right)}{\partial \varepsilon }-\frac{\partial P\left(\varepsilon ;k\right)}{\partial \varepsilon } \end{array}$$

**Figure 3:**
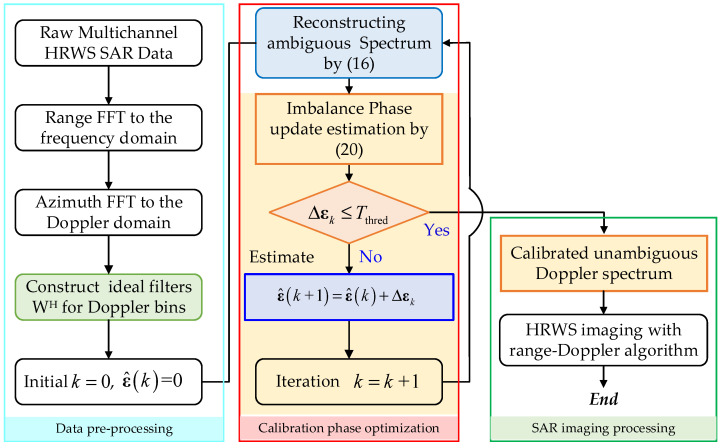
The flowchart of HRWS SAR imaging with phase calibration.

In terms of clarity, the complete calibration flowchart with the proposed maximum sharpness optimization is shown in Figure 3. One can note that the full processing flow contains three steps. The first pre-processing performs the range and azimuth FFTs on the raw multi-channel data to transfer it into the two-dimensional frequency domain. The second step calibrates the imbalance phase together with reconstructing the full Doppler spectrum without ambiguity. Finally, SAR imaging processing with the conventional range-Doppler algorithm [30] is accomplished on the reconstructed full Doppler spectrum to achieve HRWS imagery. Of course, one can also calibrate raw data with the obtained phase errors and apply adaptive beamforming for ambiguity resolving, which would take advantage of the adaptive beamformer to achieve improved image performance. The alternation of beamformers is not the key point of this paper, and in the following experiment, we perform experiments with the given flowchart. In the above deviations, the imbalance phase errors are assumed to be spatial-invariant. However, it should be noted that the extension to the range-variant imbalance calibration is straightforward by dividing the data into several range blocks and performing independent block calibration with the proposed algorithm to handle the range-dependence of array imbalance. The complete HRWS SAR processing flowchart is shown in Figure 3. The flowchart includes three import parts, namely data preprocessing, calibration phase optimization and SAR imaging processing. It is clear that the proposed algorithm is contained in the second part.

## 4. Experiments and Performance Analysis

In the following experiment, azimuth multi-channel SAR data is generated by using the real space-borne SAR of Sentinel-1 [30]. The reason for using this dataset to perform the comparative experiment is that the image contains different scenes of land with different strong buildings and open ocean areas with several strong ship targets. These ship targets provide necessary convenience for us to calculate the azimuth ambiguity suppression ratio quantitatively. The original single channel SAR data is sampled with PRF up to 1676 Hz, which is unambiguous in azimuth. In order to generate ambiguous multi-channel data, the data is re-sampled in azimuth time and down-sampled into 419 Hz to simulate a 4-channel uniform linear array data. According to the above parameters, the array baseline is about 8.427 m between two adjacent channels, and the equivalent system PRF is about 419 Hz. It should be emphasized that, in the simulated multi-channel data Doppler ambiguity number is three with the multi-channel system spatial degree of freedom is four. By the redundancy of the channel degree, we can perform the subspace projection algorithm [14] as the comparing rival for channel equalization. In order to verify the effectiveness of the proposed algorithm, imbalance phase errors between channels are randomly generated with the scope of [−π,π] and added into the multi-channel SAR data. Some important parameters of the synthetic multi-channel SAR data are listed in Table 1.

**Figure 4:**
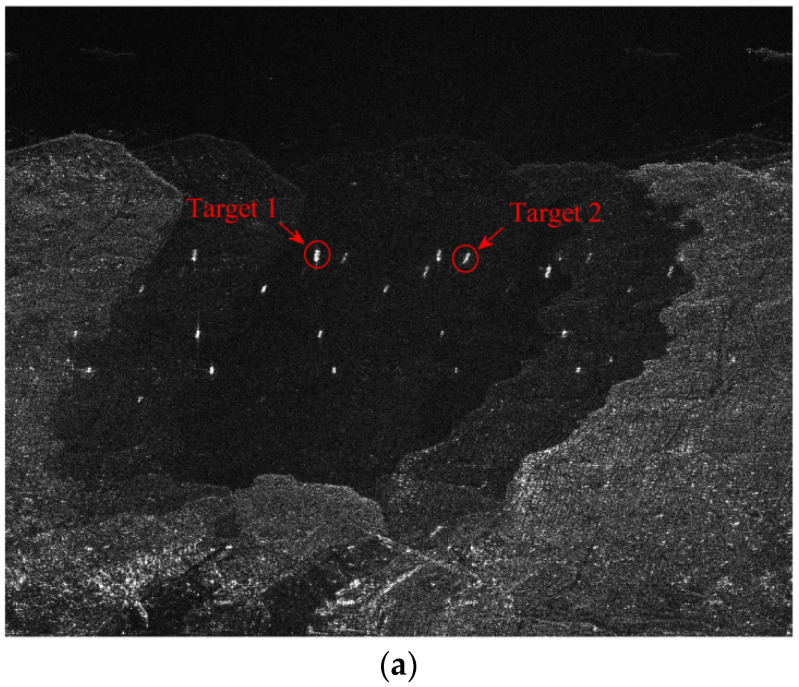

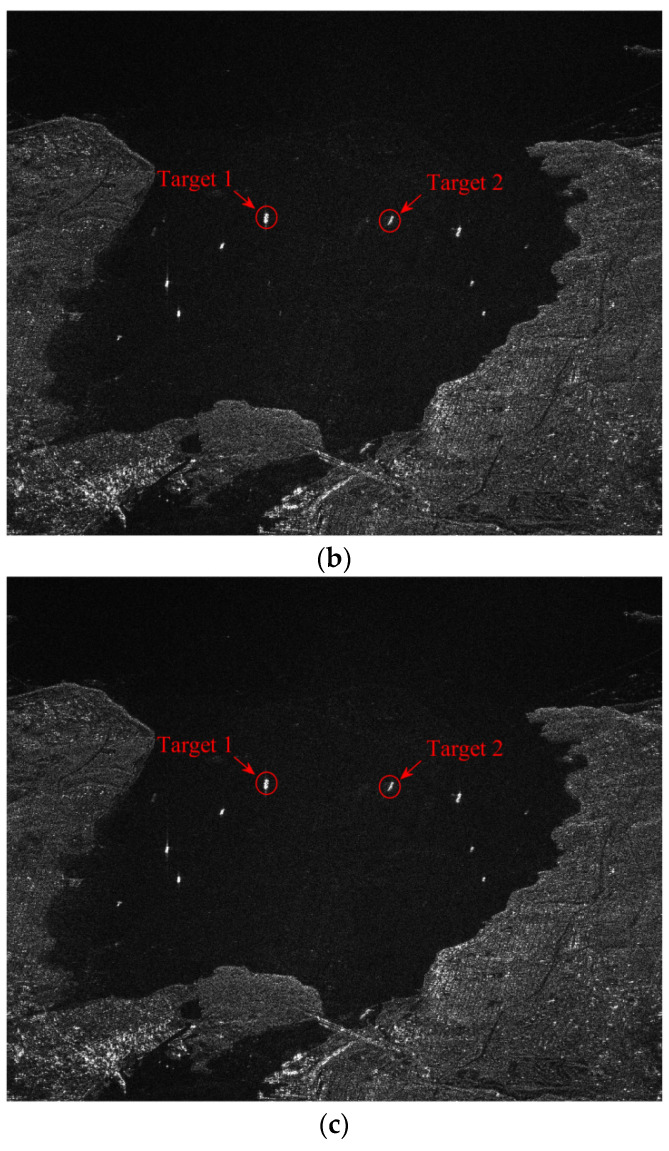
Multi-channel SAR image results with different calibrations (horizontal azimuth, vertical range). (**a**) Result without calibration. (**b**) Result with subspace projection calibration. (**c**) Result with the proposed calibration.

In the experiment, the multi-channel SAR imaging results without calibration and with phase calibration from both subspace projection algorithm and maximum Doppler spectrum sharpness algorithm are given in Figure 4a–c. As one can be seen from Figure 4a, because of the random phase errors present between channels, serious ambiguities and blurring are left in the imaging results. In the enlarged sub-images, the ambiguous components of ship targets are very obvious, which would be regarded as real ship in detection processing. On the other hand, multi-channel results from both calibrations perform well in ambiguity suppression with identical spatial filtering in the first experiment with a high SNR level. The ambiguity components present in Figure 4a are well suppressed in vision. This experiment demonstrates that when imbalance phase errors occur, calibration processing is necessary in order to achieve an optimal ambiguity suppression performance.

**Figure 5:**
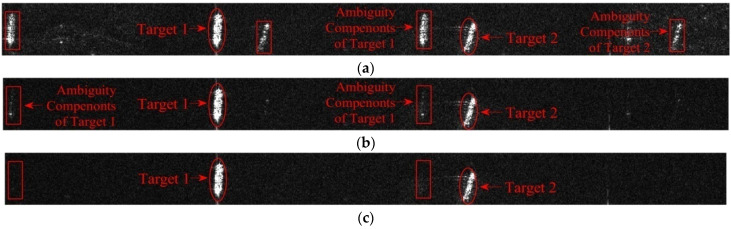
Enlarged target image results with different calibrations (horizontal azimuth, vertical range). (**a**) Result without calibration. (**b**) Result with subspace projection calibration. (**c**) Result with the proposed calibration.

In order to further show the blur suppression performance of the proposed calibration algorithm, the multi-channel SAR images of two prominent targets on ocean in Figure 4 is enlarged in Figure 5a–c correspondingly. It can be seen that the ambiguity components of ship target 1 and target 2 are greatly suppressed after the calibrations by comparing with original results in Figure 5a.

**Figure 6:**
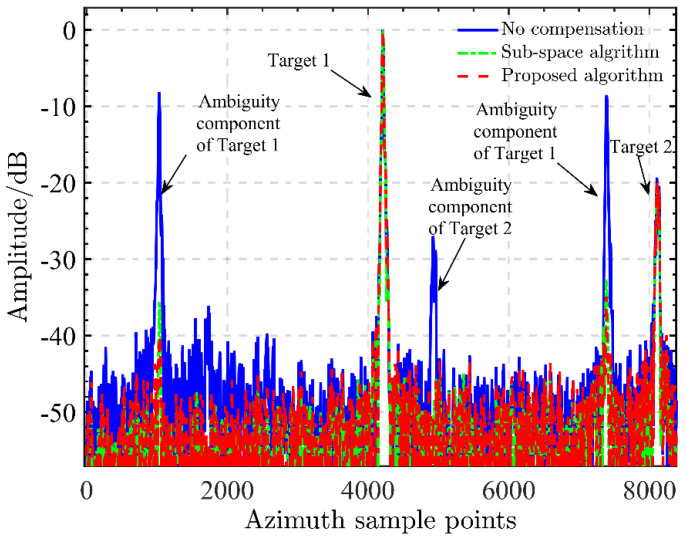
Azimuth profiles of ship targets from different calibrations.

In order to present the azimuth ambiguity suppression performance of different calibration schemes in the above experiment, the azimuth profile containing two ships defined as target 1 and target 2 is shown in Figure 6. We can note that the ambiguity components of both targets are greatly suppressed. The relative ambiguity amplitudes of targets 1 and 2 are suppressed by the proposed calibration from −9 dB and −27 dB down to about −35 dB and −50 dB, providing an improvement in the azimuth ambiguity suppression ratio up to about 28 dB. Similarly, successful calibration is also obtained by the subspace projection. It is worth noting that the relative ambiguity amplitude of target 1 is suppressed down to −32 dB, which is higher than that from the proposed calibration, reflecting the improved performance achieved by our work. Additionally, it is notable that the subspace projection calibration is less robust in the iteration number than the sharpness optimization. In real applications, the iteration number should be well set to ensure considerably optimal calibration.

**Figure 7:**
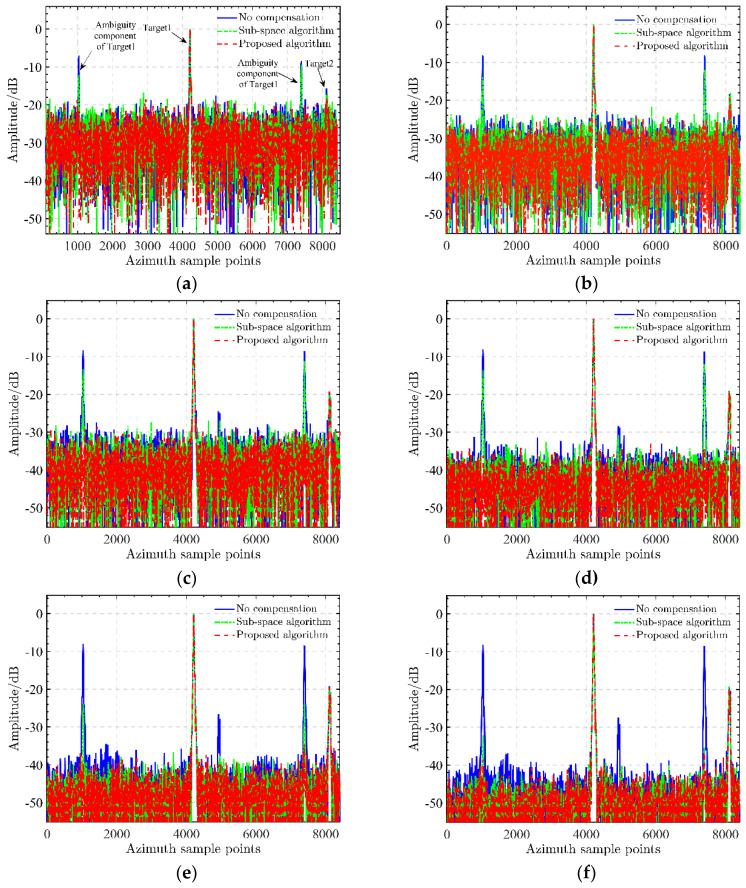

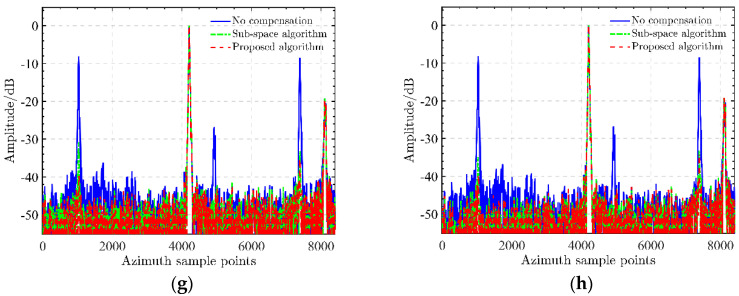
Azimuth ambiguity suppression of different calibrations under SNRs from −15 dB to 20 dB. (**a**) Results under SNR = −15 dB, (**b**) Results under SNR = −10 dB, (**c**) Results under SNR = −5 dB, (**d**) Results under SNR = 0 dB, (**e**) Results under SNR = 5 dB, (**f**) Results under SNR = 10 dB, (**g**) Results under SNR = 15 dB, (h) Results under SNR = 20 dB.

**Figure 8:**
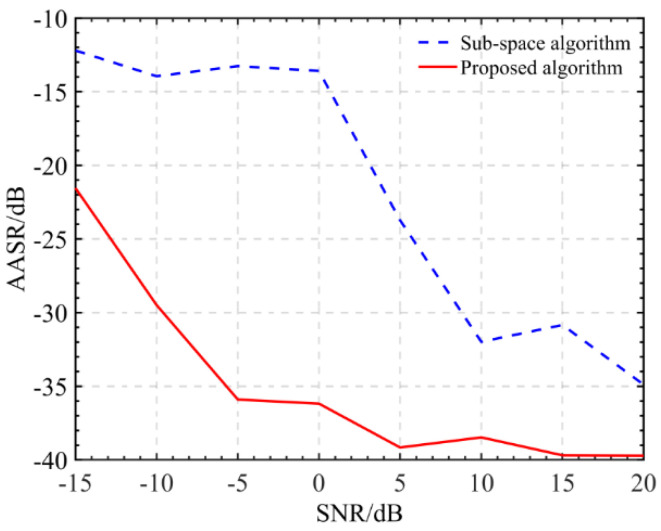
AASRs with different SNRs.

In this section, we would like to further confirm the robustness of the proposed sharpness optimization calibration to strong noise. A channel calibration experiment is carried out by adding different noise into the multi-channel data with phase imbalance. Datasets with different signal-to-noise ratios (SNR) from −15 dB~20 dB are generated by adding complex white Gaussian noise to the original multi-channel data implemented by the AWGN function of MATLAB 2020a software. The experimental results are shown in Figure 7a–h. As you can see from the picture, in the cases of low SNR, such as SNR down to 0 dB, the AASR of the typical target 1 from subspace projection algorithm is about −15 dB. On the other hand, with an increase in SNR setting, such as SNR up to 5 dB, the calibration performance of the subspace projection algorithm is gradually guaranteed, as the AASR usually reaches up to −20 dB. When SNR = 20 dB, the AASR from the subspace algorithm is optimal, down to about −35 dB. Clearly, the noise effect on the subspace projection algorithm for calibration is demonstrated. We can conclude that a considerably high SNR is the fundamental condition for the success of the subspace projection algorithm. On the other hand, the sharpness optimization calibration algorithm is generally robust to strong noise. This is because it makes use of all signals to solve the phase error estimation. Even in the scenarios under low SNR, ambiguity suppression with Doppler spectrum sharpness optimization maintains its optimal performance. In the experiment, with varying SNRs, the AASRs obtained by the proposed algorithm remained stable and optimally maintained down to −25 dB. For clarity of the comparison, we provide the AASRs with varying SNR from −15 dB to 20 dB, from which we can note the advantage of the proposed calibration in handling strong noise over subspace calibration. To illustrate the excellent performance of the proposed algorithm, the AASRs of different algorithms with different SNRs are shown in Figure 8. In the experiment, with varying SNRs, the AASRs obtained by the proposed algorithm remained stable and optimally maintained down to −25 dB. For clarity of the comparison, we provide the AASRs with varying SNR from −15 dB to 20 dB, from which we can note the advantage of the proposed calibration in handling strong noise over subspace calibration. From these comparing results, one can conclude that the proposed algorithm has robustness in front of strong noise, which is suitable for calibrating a wide range of multi-channel SAR imagery applications.

## 5. Conclusions

Imbalance calibration is an important task for HRWS unambiguous SAR imaging with multi-channel SAR. In this paper, we proposed a phase calibration method with maximum sharpening of the reconstructed Doppler spectrum. The sharpness maximization optimization was developed to bridge the imbalance phase estimation and solve the optimization with a gradient-based algorithm. Without requirement on additional spatial freedom degrees, the proposed calibration provides reliable phase correction performance in both precision and efficiency aspects. Synthetic experiments are used to confirm the validity of the calibration by comparing conventional algorithms, which confirms the superiorities of the proposed calibration. We have to emphasize that, range- and azimuth-variant channel imbalance would be present in real HRWS SAR applications, which induces many difficulties for channel calibration with high efficiency and robustness. These interesting problems are still open in HRWS SAR research and left to future work.

## Figures and Tables

**Table 1:** System parameters.

Wavelength	Pulse Width	Sample Rate	Bandwidth	Azimuth Points	Range Points	PRF	Equivalent Velocity
0.056 m	41.750 μs	32.3 MHz	30.1 MHz	2048	2048	1257 Hz	7062 m/s

## Data Availability

Not applicable.
